# Effect of Culture Conditions of *Lophocereus marginatus* Endophytic Fungi on Yield and Anticancer and Antioxidant Activities

**DOI:** 10.3390/ijerph20053948

**Published:** 2023-02-23

**Authors:** Jesica María Ramírez-Villalobos, Ricardo Gomez-Flores, Priscilla Viridiana Velázquez-Flores, Karla Selene Morán-Santibáñez, Patricia Tamez-Guerra, Orquídea Pérez-González, Myriam Angélica de la Garza-Ramos, Cristina Rodríguez-Padilla, César Iván Romo-Sáenz

**Affiliations:** 1 Departamento de Microbiología e Inmunología, Facultad de Ciencias Biológicas, Universidad Autónoma de Nuevo León, San Nicolás de los Garza 66455, Mexico; 2 Centro de Investigación y Desarrollo en Ciencias de la Salud, Facultad de Odontología, Universidad Autónoma de Nuevo León Dr. Eduardo Aguirre Pequeño y Silao S/N, Colonia Mitras Centro, Monterrey 64460, Mexico; 3 Universidad Emiliano Zapata, Avenida Rodrigo Gómez, Sector Heroico S/N, Monterrey 64260, Mexico

**Keywords:** fermentation, *Metarhizium anisopliae*, *Aspergillus versicolor*, lymphoma, antitumor, endophyte

## Abstract

Culture conditions affect the production of secondary metabolites in endophytic fungi. Therefore, the aim of the present study was to evaluate the yield and anticancer and antioxidant activity of endophytic fungi extracts from the cactus *Lophocereus marginatus*, under different culture conditions. The strains *Penicillium citrinum*, *Aspergillus versicolor*, *Metarhizium anisopliae,* and *Cladosporium* sp. were fermented in different culture media (potato dextrose agar, Czapeck broth, and malt broth), types of inoculums (spore or mycelium), and shaking conditions (150 rpm or static) for one week. Methanol extracts were obtained from mycelia, which was followed by determining their yields and evaluating their effect on L5178Y-R murine lymphoma cells growth and human peripheral blood mononuclear cells (PBMCs) viability, using the 3-[4,5dimethylthiazol-2-yl]2,5-diphenyl tetrazolium bromide reduction colorimetric assay. In addition, antioxidant activity was determined by the 2,2-diphenyl-1-picrylhydrazyl test. We determined the half-maximal inhibitory concentration (IC_50_) values of tumor cell growth inhibition, the selectivity index (SI), and the antioxidant activity, as compared with the healthy cells control. The best yields were obtained with the Czapeck broth medium in all the evaluated strains, reaching values of 50.3%. Of the 48 extracts evaluated, only seven significantly (*p* < 0.01) inhibited tumor cell growth (IC_50_ < 250 µg/mL). *A. versicolor* extract showed the highest anticancer activity, after culturing spores (IC_50_ = 49.62 µg/mL; SI = 15.8) or mycelium (IC_50_ = 69.67 µg/mL; SI = 12.2) in malt broth, under static conditions. Extracts did not present significant antioxidant activity. In conclusion, we showed that culture conditions influenced the anticancer activity of *L. marginatus* endophytic fungi.

## 1. Introduction

Among endophytic microorganisms, fungi represent one of the most important sources of bioactive compounds. They have developed the potential to produce compounds similar to those of their hosts [1], novel metabolites [2], and transform natural products by changing their structures and bioactivities [3]. Various classes of metabolites with immunomodulatory, antimicrobial, anticancer, and antioxidant activities have been reported with the potential to be biotechnologically exploited [4]. However, for the production of secondary metabolites from endophytic fungi to be economically viable, it is necessary to increase the biological activity of the fungal strains as well as the yield of bioactive compounds [5].

One of the simplest and widely used strategies to increase biological activity is the modification of culture conditions [6], since it allows to obtain different metabolite profiles in response to the environment [7]. We can control physical factors such as agitation and temperature, chemical factors such as the composition, pH, or salinity of the culture medium, and biological elements such as the type of inoculum (spore or vegetative) or co-culture with other microorganisms [8]. Promising results have been obtained by implementing this strategy in fungal species such as *Penicillium citrinum* [9], *Aspergillus versicolor* [10], *Metarhizium anisopliae* [11], and *Cladosporium* sp. [12]. Despite these findings, the extrapolation of culture conditions to other strains of the same species is not always successful due to the genetic diversity that exists among the gene clusters involved in the metabolic pathways for the production of secondary metabolites. Consequently, the biological activity as well as the response to the environment may be different [13]. Therefore, it is necessary to continue investigating the factors that influence the production of metabolites in these species.

We have previously reported the anticancer activity of the endophytic fungal strains *P. citrinum* (strain PME-H002), *A. versicolor* (strain PME-H005), *M. anisopliae* (strain PME-H007), and *Cladosporium* sp. (strain PME-H008) isolated from the cactus *Lophocereus marginatus* [Cactaceae] [14]. However, the effect of culture conditions on their biological activity has not been yet evaluated. Therefore, the aim of the present study was to evaluate the effect of the culture medium, type of inoculation, and agitation, on the yield, and anticancer and antioxidant activities of methanol extracts of endophytic fungi from *L. marginatus*.

## 2. Materials and Methods

### 2.1. Fungal Strains

We used the following strains of endophytic fungi isolated from *L*. *marginatus*: *P. citrinum* (strain PME-H002), *A. versicolor* (strain PME-H005), *M. anisopliae* (strain PME-H007), and *Cladosporium* sp. (strain PME-H008), which were provided by the Laboratorio de Inmunología y Virología in Facultad de Ciencias Biológicas at Universidad Autónoma de Nuevo León, México.

### 2.2. Fungi Fermentation and Extract Preparation

Fungal strains were activated on potato dextrose agar (PDA; Difco Laboratories, Detroit, MI) for 7 to 12 d at 28 °C ± 2 °C. To evaluate the production of metabolites, the following culture media were used: potato dextrose broth (PDB; Difco Laboratories) at pH 5.7 ± 0.2, Czapeck broth (CKB; 30 g/L sucrose, 2 g/L NaNO_3_, 1 g/L KH_2_PO_4_, 0.5 g/L MgSO_4_, 0.5 g/L KCl, and 0.01 g/L FeSO_4_) at pH 7.3 ± 0.2, and malt broth (MB) (13 g/L maltose, 5.5 g/L casein peptone, and 0.5 g/L yeast extract) at pH 4.7 ± 0.2, containing 10,000 U/mL penicillin and 10 mg/mL streptomycin (Difco Laboratories). Different types of inoculums and shaking conditions were also implemented, for which 250 mL flasks containing 125 mL of culture media were inoculated with a 0.8 cm^2^ fragment of fresh mycelium or with spores at a concentration of 1 × 10^6^ spores/mL. Inoculated flasks were fermented for one week under shaking conditions (150 rpm) or kept static at room temperature (28 °C ± 2 °C), using three replicate determinations per treatment (Table 1). Next, biomass was separated from by filtration and left to dry at 40 °C, which was followed by extraction by maceration with methanol at a 1:20 biomass:methanol ratio. Solvent was then removed with a Buchi R-3000 rotary evaporator (Büchi Labortechnic, Postfach, Switzerland). Extraction yield was calculated using the following formula: yield = (grams of dry extract/grams of biomass) (100). Dry extracts were reconstituted with dimethyl sulfoxide (Sigma-Aldrich, St. Louis, MO) at a concentration of 25 mg/mL.

### 2.3. Cell Cultures

We used the murine lymphoma cell line L5178Y-R (ATCC CRL-1722), as well as human peripheral blood mononuclear cells (PBMC) as the control group, which were obtained from 20 to 30 mL of blood samples from healthy volunteer donors, using Ficoll–Paque Plus (GE Healthcare Life Sciences, Pittsburgh, PA) to separate white cells. Cells were maintained in RPMI-1640 medium (Life Technologies, Rockville, MD), supplemented with 10% fetal bovine serum (FBS; Life Technologies) and 1% antibiotic-antifungal solution (Life Technologies), and incubated at 37 °C in a 5% CO_2_ atmosphere in air.

### 2.4. L5178Y-R and PBMC Growth Inhibition Assay

L5178Y-R lymphoma cells (1 × 10^4^ cells/well) and PBMC (1 × 10^5^ cells/well) were incubated in 96-well plates (Corning Incorporated, Corning, NY) in the presence or absence of 15 to 250 µg/mL of fungal extracts for 48 h at 37 °C in an atmosphere of 5% CO_2_ in air. Growth inhibition was then evaluated by the 3-(3,4-dimethylthiazol-2-yl)-2,5-diphenyltetrazolium bromide (MTT; Invitrogen, Carlsbad, CA) reduction colorimetric assay [15], adding 15 µL/well (0.5 mg/mL final concentration) and incubating at 37 °C for 4 h. Formazan crystals were dissolved with DMSO and optical densities (OD) were measured at 570 nm in a Multiskan GO microplate reader (Thermo Fisher Scientific, Rockford, IL). Percentage growth inhibition was calculated by comparing the OD of treated cells with that of untreated cells, using the following formula: % growth inhibition = 100 − [(OD of extract treated cells/OD of untreated cells) (100)]. As a positive control, 0.05 µg/mL vincristine sulfate (Kocak Pharma, Istanbul, Turkey) was used. The concentration at which the extracts cause 50% growth inhibition (IC_50_) was calculated using a non-linear regression analysis, and with these data, the selectivity index (SI) was calculated using the following formula: IC_50_ of normal cells/IC_50_ of tumor cells, which was only calculated for extracts that presented IC_50_ below 250 µg/mL against lymphoma cells.

### 2.5. Antioxidant Activity

Antioxidant activity of fungal extracts was determined by the 2,2-diphenyl-1-picrylhydrazyl (DPPH) test (Santa Cruz Biotechnology, Santa Cruz, CA) [16]. Only extracts that showed IC_50_ below 250 µg/mL were analyzed. Different concentrations (15.625 µg/mL to 500 µg/mL) of the extract were added to 96-well plates in a final volume of 100 µL, after which 100 µL of 88 µM DPPH diluted in methanol were added to the wells and homogenized. Plates were then incubated in darkness for 30 min, after which they were read in a microplate reader (Thermo Fisher Scientific) at 517 nm. We used 50 µg/mL ascorbic acid as a positive control and DMSO (blank) as a negative control. The antioxidant activity was expressed as percentage inhibition and was determined with the following formula: % inhibition = [(OD blank − OD sample)/OD blank] × 100. IC_50_ values were calculated with the data obtained. The level of activity was classified as weak (IC_50_ > 1000 µg/mL), moderate (IC_50_ = 200 µg/mL to 1000 µg/mL), and high (IC_50_ < 200 µg/mL) [17].

### 2.6. Statistical Analysis

Results were expressed as mean ± SD of three replicates determination from three independent experiments. Normal distribution of data was analyzed by the D’Agostino–Pearson normality test. For the statistical analysis, we used P < 0.01. The percentages were transformed with the arcsine function for the parametric tests. The one-way ANOVA, followed by Tukey’s comparison of means and the Kruskal–Wallis test, followed by the Dunnet’s test were used. IC_50_ values were reported with 95% confidence intervals. Analyses were performed using the Graph Pad Prism 7 software (GraphPad Software Inc., San Diego, CA, USA).

## 3. Results

### 3.1. Extract Yields

**Figure 1:**
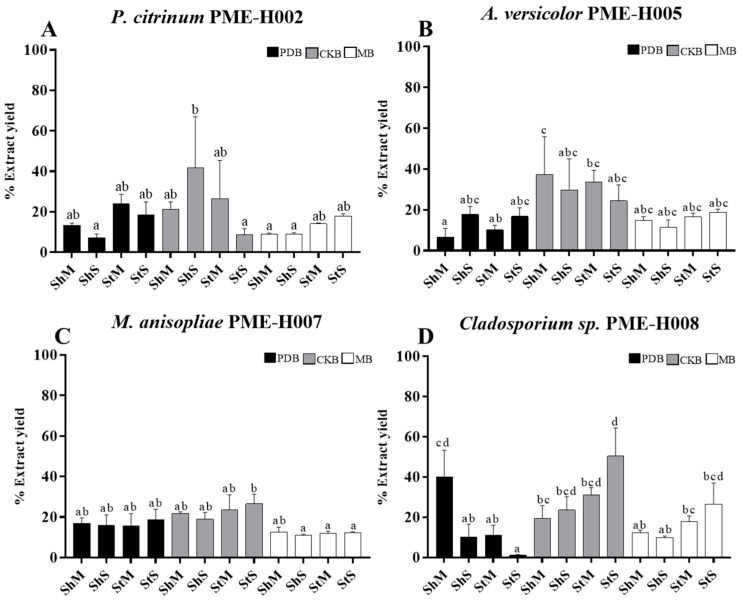
Yield of methanol extracts from endophytic fungal strains under different culture conditions. (**A**) *P. citrinum* PME-H002. (**B**). *A. versicolor* PME-H005. (**C**) *M. anisopliae* PME-H007. (**D**) *Cladosporium* sp. PME-H008. Data represent the mean ± SD of three replicate determinations from three independent experiments. Different letters indicate significant differences (*p* < 0.01), using the one-way ANOVA. Sh, shaking; St, static; M, mycelium; and S, spore.

Fungal strains were fermented in PDB, CKB, and MB culture media, using the following conditions: shaking + mycelium fragment inoculum (ShM), shaking + 1 × 10^6^ spores/mL inoculum (ShS), static + mycelium fragment inoculum (StM), and static + 1 × 10^6^ spores/mL (StS). It was found that the best yields were obtained with CKB for all the evaluated strains. We observed the highest yield (41.7%) with *P. citrinum* PME-H002 strain, after culturing under agitation and inoculated with spores (ShS) in CKB medium, as compared with PDB (*p* = 0.002) and MB (*p* = 0.006), under the same conditions (Figure 1A), whereas the highest yield (37.4%) with the *A. versicolor* PME-H005 strain was obtained after shaking and mycelium fragment (ShM) inoculum in the CKB medium (Figure 1B). Furthermore, the highest yield (26.6%) with the *M. anisopliae* PME-H007 strain was obtained by inoculating spores under static condition (StS) in the CKB culture medium, showing significant differences (P < 0.05) compared with fermentation in MB (Figure 1C), whereas the highest yield (50.3%) with the *Cladosporium sp*. PME-H008 strain was obtained after inoculating with spores without shaking (StS) in the CKB medium. However, we obtained a high 40.02% yield after shaking and mycelium fragment inoculation (ShM) in PDB culture medium (Figure 1D).

### 3.2. Cell Growth Inhibition

**Figure 2:**
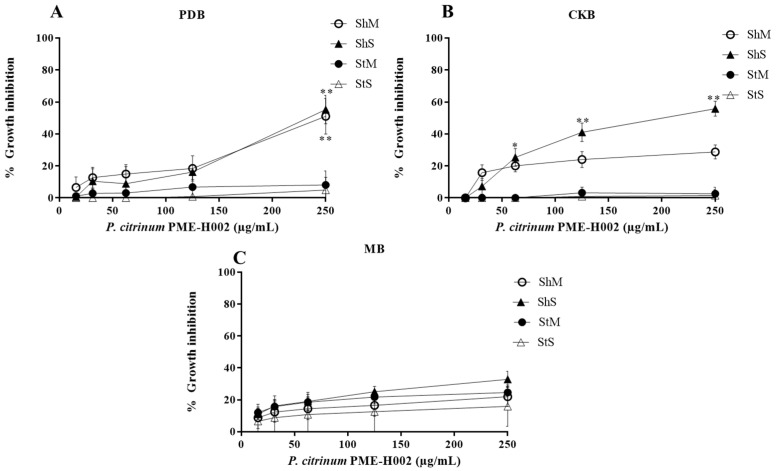
Percentage growth inhibition of *P. citrinum* PME-H002 strain methanol extracts against L5178Y-R cells in different media and culture conditions. (**A**) PDB, (**B**) CKB, and (**C**) MB. Data represent the mean ± SD of three replicate determinations from three independent experiments. * *p* < 0.05, ** *p* < 0.01, as compared with the untreated control using the Kruskal–Wallis test. Sh, shaking; St, static; M, mycelium; S, spore.

**Figure 3:**
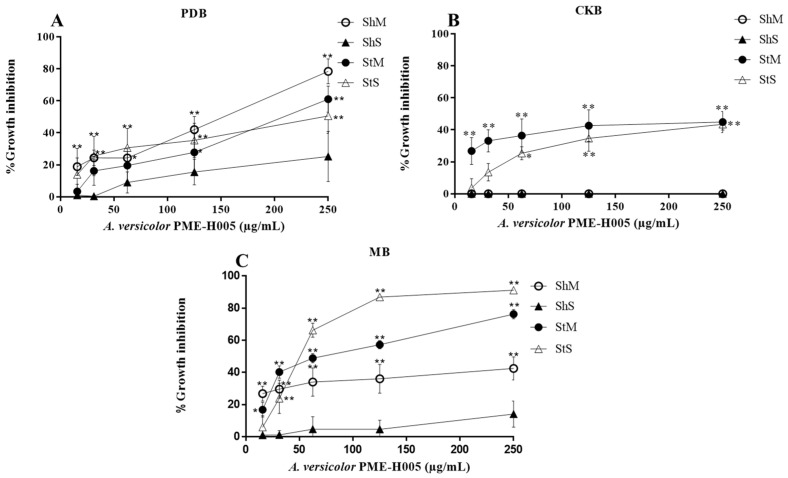
Percentage growth inhibition of *A. versicolor* PME-H005 strain methanol extracts against L5178Y-R cells in different media and culture conditions. (**A**) PDB, (**B**) CKB, and (**C**) MB. Data represent the mean ± SD of three replicate determinations from three independent experiments. * *p* < 0.05, ** *p* < 0.01, as compared with the untreated control, using the Kruskal–Wallis test. Sh, shaking; St, static; M, mycelium; S, spore.

**Figure 4:**
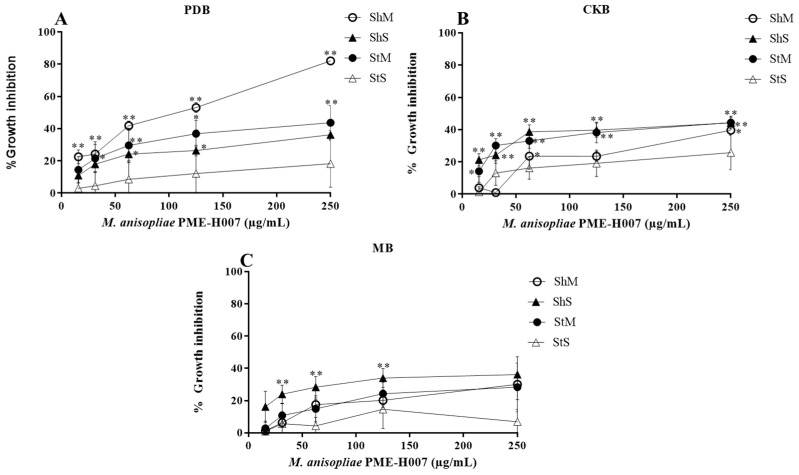
Percentage growth inhibition of *M. anisopliae* PME-H007 strain methanol extracts against L5178Y-R cells in different media and culture conditions. (**A**) PDB, (**B**) CKB, and (**C**) MB. Data represent the mean ± SD of three replicate determinations from three independent experiments. * *p* < 0.05, ** *p* < 0.01, as compared with the untreated control, using the Kruskal–Wallis test. Sh, shaking; St, static; M, mycelium; S, spore.

**Figure 5:**
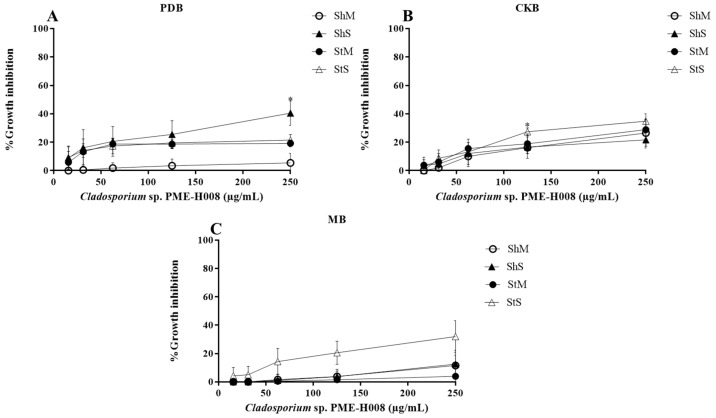
Percentage growth inhibition of *Cladosporium* sp. PME-H008 strain methanol extracts against L5178Y-R cells in different media and culture conditions. (**A**) PDB, (**B**) CKB, and (**C**) MB. Data represent the mean ± SD of three replicate determinations from three independent experiments. * *p* < 0.05, ** *p* < 0.01, as compared with the untreated control, using the Kruskal–Wallis test. Sh, shaking; St, static; M, mycelium; S, spore.

We used 15 to 250 µg/mL methanol extracts of *L. marginatus* endophytic fungi to determine L5178Y-R lymphoma cells growth inhibition and IC_50_. Extracts significantly (*p* < 0.05) inhibited tumor cells growth in a concentration-dependent manner (Figure 2, Figure 3, Figure 4 and Figure 5). At the highest concentration (250 µg/mL), the strain with the highest activity (*p* < 0.01) was *A. versicolor* PME-H005 with 91.1% growth inhibition when grown in MB inoculated with spores without shaking (StS) (Figure 3C). Significant growth inhibition (IC_50_ < 250 µg/mL) was observed with *P. citrinum* PME-H002 (Figure 2A,B), *A. versicolor* PME-H005 (Figure 3A,C), and *M. anisopliae* PME-H007 (Figure 4A), and IC_50_ values of the extracts were determined (Table 2). However, none of the culture conditions of *Cladosporium* sp. PME-H008 strain increased the tumor IC_50_ (Figure 5). Extracts that showed IC_50_ below 250 µg/mL against L5178Y-R cells (7 out of 48 extracts) were selected to be evaluated against normal PBMC and determined the SI (Table 2). These extracts presented IC_50_ values higher than 250 µg/mL. *P. citrinum* PME-H002 strain caused an IC_50_ of 8993 ± 0.2 µg/mL on PBMC after culturing in CKB under agitation and inoculating with spores. We used the IC_50_ of tumor and normal cells growth inhibition to calculate the SI, finding that the extracts showed values from 10.5 to 82.7, where the strain that presented the highest value was *A. versicolor* PME-H005 with an SI of 82.7 after culturing in PDB and inoculating with mycelium in agitation, which was followed by *P. citrinum* PME-H002 strain with an SI of 48.4 in CKB in agitation and inoculating with spores. Vincristine positive control caused 85% lymphoma cells and 11.2% PBMC growth inhibition.

### 3.3. Antioxidant Activity

The antioxidant activity of methanol extracts of *L. marginatus* endophytic fungi was evaluated by the DPPH test. It was found that the *P. citrinum* PME-H002 strain was the only one with moderate antioxidant activity (IC_50_ = 200 to 1000 µg/mL), with an IC_50_ value of 988.4 ± 1.4 µg/mL, after culturing in PDB at 150 rpm and inoculating with mycelium (Table 3). The rest of the extracts evaluated did not show significant antioxidant activity (IC_50_ higher than 1000 µg/mL). The highest antioxidant activity was obtained with ascorbic acid (positive control) with an IC_50_ value of 7.1 ± 1.1 µg/mL.

## 4. Discussion

In this study, we demonstrated the influence of culture conditions on yield and anticancer and antioxidant activities of endophytic fungi from *L. marginatus*. It was shown that the culture medium, type of inoculum, and agitation were factors that significantly affected the extraction yield and the selective anticancer activity against the murine lymphoma cell line L5178Y-R of the evaluated fungal strain extracts.

Modification of parameters in the fermentation process increased the growth and yield of fungal strains [18]. However, this is not always related to the increase in biological activity [19]. In our study, the use of Czapek medium increased the yield of the evaluated strains but only the strain *P. citrinum* PME-H002 significantly augmented tumor cell growth inhibition.

Endophytic fungi under laboratory conditions are usually very sensitive to nutrient alterations [6]; adaptation to these changes induces the expression of genes related to the biosynthesis of secondary metabolites, which is responsible for biological activity [7]. Therefore, a fungal strain may produce different amounts and diversity of bioactive compounds, depending on the nutrients present in the medium [20]. In the present study, three culture media were evaluated that differ in the source of carbon and nitrogen, which are the major components that generally influence metabolite production [21]. *P. citrinum* PME-H002 strain anticancer activity increased in CKB, followed by PDB, which agrees with other studies using penicillocitrin, benzoic acid, and secalonic acid obtained from *P. citrinum* cultures in PDB [22]. In the case of the *A. versicolor* PME-H005 strain, we obtained significant anticancer activity culturing in MB, which is also in agreement with other studies where malt was included in the culture medium to obtain *A. versicolor* compounds such as flutamide C and F, sterigmatocystin, averanthin, and nidurufin with cytotoxic activity against tumor lung, skin, and ovarian tumor cell lines [23,24], whereas for the *M. anisopliae* PME-H007 strain, it was shown that PDB was the only one that significantly increased anticancer activity. El-Maali et al. [25] reported the production of the anticancer compound taxol by *M. anisopliae* (AUMC 5130) in PDB, whereas the anticancer agents swainsonin and destruxins were obtained from *M. anisopliae* grown in CKB and other media with maltose, glucose, and starch as carbon sources [11,26,27].

Agitation is another important factor in fermentation processes, since it supplies oxygen to the system, affects heat transfer, and generates cell morphological changes [28], which may affect biochemical reactions and activate genes for the production of different secondary metabolites [7]. In our study, *P. citrinum* PME-H002 and *M. anisopliae* PME-H007 strains increased their activities when cultured at 150 rpm. It has been shown that agitation is necessary for *M. anisopliae* to produce compounds such as swainsonine [29]. However, *P. citrinum* metabolites with anticancer potential such as citriquinochroman have been obtained under static conditions [30] or pencitrin and pencitrinol have been obtained under agitation [31].

It is known that the type of inoculum (spore or vegetative) affects the production of fungal metabolites. We found that the *P. citrinum* PME-H002 strain showed significant anticancer activity after inoculating spores that may be due to the germination process in which the germ tube develops, which spreads through the absorption and metabolism of nutrients [8], affecting the production of final secondary metabolites [32]. Inoculation with mycelium favored the production of bioactive metabolites in *M. anisopliae* PME-H007. This type of inoculum may decrease the latency phase, unlike inoculation with spores [33], which speeds up the process for obtaining compounds responsible for the anticancer activity.

The requirement for new drugs that are highly efficient and low in toxicity for the patient is increasing [18]. Therefore, the selectivity index (SI) is a very useful parameter to discard compounds or extracts that show adverse effects on normal cells in in vitro evaluations [34]. Our results demonstrated that the evaluated extracts in PBMC showed selective toxicity toward lymphoma cells with SI values >10, which according to Peña-Morán et al. [35] indicates that the extracts may be considered selective and are candidates for future research.

We did not observe any relevant antioxidant activity (IC_50_ < 200 µg/mL) of the extracts. In contrast, Chandra and Arora [36] found that the antioxidant activity of *P. citrinum* increased by modifying the components of CKB, associating such an activity with the presence of phenolic compounds. Various reports have shown the potential of *A. versicolor* to produce antioxidant compounds such as extracellular polysaccharides [37], L-glutaminases [38], pyrone derivative [39], and polyketides [40]. Abdel-Wareth et al. [10] reported that ethyl acetate and acetone extracts from *M. anisopliae* possessed antioxidant activity when evaluated by the phosphomolybdenum method. The lack of antioxidant activity of our fungal strains may be due to various factors such as culture conditions, type of extraction (biomass or supernatant), solvent used [41], or the absence of genes involved in the production of antioxidant compounds [5]. Strains of the same species may differ in their potential to produce secondary metabolites, which is the reason to evaluate different culture conditions to increase the performance and biological activity of fungal strains [42].

## 5. Conclusions

This study provides relevant information for obtaining bioactive compounds of endophytic fungi from *L. marginatus*. Particularly, *A. versicolor* PME-H005 strain excelled for its selective anticancer activity against L5178Y-R cells, after culturing in MB, under static conditions. Further research is required to elucidate the bioactive compounds responsible for the anticancer activity as well as their mechanism of action.

## Figures and Tables

**Table 1:** Culture media and experimental test conditions used to evaluate the level of production of metabolites.

Culture Media		Experimental Test Conditions	
Medium	Description	Test	Description
PDB	Potato dextrose broth	ShM	Shaking + Mycelium fragment inoculum
CKB	Czapeck broth	ShS	Shaking + 1 × 10^6^ Spores/mL inoculum
MB	Malt broth	StM	Static + Mycelium fragment inoculum
		StS	Static + 1 × 10^6^ Spores/mL inoculum

**Table 2:** IC_50_ (µg/mL) and SI of L5178Y-R cells and PBMC treated with *L. marginatus* fungal endophyte methanol extracts from different culture conditions.

Strain	Culture Medium	Shaking	Inoculum	L5178Y-R IC_50_	PBMC IC_50_	SI ^a^
*P. citrinum* PME-H002	PDB	150 rpm	Spores	234 ± 1.5 c	2961 ± 0.4 a	12.6
	CKB	150 rpm	Spores	185.8 ± 1.5 bc	8993 ± 0.2 a	48.4
*A. versicolor* PME-H005	PDB	150 rpm	Mycelium	123.5 ± 1.3 ab	4334 ± 0.4 a	35
	PDB	Static	Mycelium	203.2 ± 1.3 bc	16811 ± 0.2 a	82.7
	MB	Static	Mycelium	69.67 ± 1.5 a	851.4 ± 0.7 a	12.2
	MB	Static	Spores	49.62 ± 1.8 a	784.1 ± 0.9 a	15.8
*M. anisopliae* ME-H007	PDB	150 rpm	Mycelium	84.56 ± 1.5 a	894.8 ± 0.9 a	10.5

^a^ SI = IC_50_ PBMC/IC_50_ L5178Y-R. Values with different letters within columns are significantly (*p* < 0.01) different, using the one-way ANOVA.

**Table 3:** IC_50_ (µg/mL) of antioxidant activity of endophytic fungi methanol extracts with anticancer activity.

Strain	Culture Medium	Shaking	Inoculum	DPPH IC_50_	Activity ^b^
*P. citrinum* PME-H002	PDB	150 rpm	Spores	5792 ± 0.2 b	Weak
	CKB	150 rpm	Spores	988.4 ± 1.4 b	Moderate
*A. versicolor* PME-H005	PDB	150 rpm	Mycelium	1163 ± 0.8 b	Weak
	PDB	Static	Mycelium	29341 ± 0.07 b	Weak
	MB	Static	Mycelium	1874 ± 0.8 b	Weak
	MB	Static	Spores	1935 ± 0.9 b	Weak
*M. anisopliae* ME-H007	PDB	150 rpm	Mycelium	3647 ± 0.7 b	Weak
Ascorbic acid	NA ^a^	NA ^a^	NA ^a^	7.1 ± 1.1 a	High

^a^ NA = Not applicable. ^b^ Weak = IC_50_ > 1000 µg/mL; moderate = IC_50_ 200 to 1000 µg/mL; and high = IC_50_ < 200 µg/mL. Values with different letters within columns are significantly (P < 0.01) different, using the one-way ANOVA.

## Data Availability

The datasets generated and/or analyzed during the present study are available from the corresponding author on reasonable request.
